# Pathogenesis of Fungal and Bacterial Microbes

**DOI:** 10.3390/pathogens9080602

**Published:** 2020-07-23

**Authors:** Jennifer Geddes-McAlister

**Affiliations:** Molecular and Cellular Biology Department, University of Guelph, Guelph, ON N1H 1N8, Canada; jgeddesm@uoguelph.ca

**Keywords:** bacterial pathogenesis, fungal pathogenesis, host-pathogen interactions, host defense, virulence factors, systems biology, antimicrobials

## Abstract

The pathogenesis of fungal and bacterial microbes is a complex process involving distinct parameters, including virulence factors, nutrient sensing and availability, microbial signals, as well as host status and defense responses. Defining pathogenesis improves our understanding of how an organism causes diseases and provides insight into novel prospects to combat infection. The effects of pathogenic microbes have significant impact on diverse sectors, including health, agriculture, and economics, underscoring their immense importance in society. Articles in this Special Issue address unique aspects of microbial pathogenesis by exploring interactions between host and pathogen during infection, defining inflammatory immune responses, profiling the importance of essential microbial structures associated with virulence, and outlining critical considerations driving complex diseases.

Fungal and bacterial pathogens represent a significant threat to human, animal, and environmental health. For example, the formation of hybrids between fungal species can complicate treatment strategies and influence the development of antifungal resistance [1]. Dong et al. assessed the genetic stability of hybrids between *Cryptococcus neoformans* and *Cryptococcus deneoformans*, fungal pathogens found ubiquitously in the environment and primarily causing infection in immunocompromised individuals, using mutation accumulation lines of a diploid [2]. The authors quantify differences in loss-of-heterozygosity between standard and antifungal stress (e.g., fluconazole treatment) conditions. The study concludes that hybrids in *C. neoformans* species complex are generally stable; however, during encounter with an antifungal, rapid adaptation to environmental stresses through loss-of-heterozygosity and gene duplication can occur.

To influence the outcome of fungal infections, microbes elaborate diverse virulence factors (e.g., polysaccharide capsule), secrete extracellular enzymes (e.g., proteases), and produce toxins (e.g., mycotoxins). Eranthodi et al. explored the effectiveness of the cyclohexadepsipeptide enniatins mycotoxin produced by the fungal pathogen, *Fusarium avenaceum* on crops outside its typical reservoir [3]. Here, the non-traditional hosts of pea roots and durum wheat spikes were assessed for necrosis, as commonly observed in potato tubers, using *F. avenaceum* mutant strains with an *ENNIATIN SYNTHASE* 1 (*ESYN*1) disruption or overexpression. The authors report no changes in disease symptoms or virulence caused by the selected enniatin on the alternative hosts, supporting a host-specific role of the mycotoxin for moderating disease severity.

To prevent infection from microbial pathogens, the host presents many protective barriers (e.g., epithelial layer, pH, antimicrobial peptides). Upon breach of such barriers, the blood brain barrier presents another hurdle of protection for the central nervous system against microbial invasion. Liu et al. explored the impact of blood brain barrier disruption in the development of bacterial meningitis [4]. Using in vitro and in vivo models of *Escherichia coli* meningitis, the authors report upregulation of angiopoietin-like 4 (a fasting-induced inhibitor of lipoprotein lipase and regulator of plasma triglyceride metabolism), in brain microvascular endothelial cells, leading to disruption of the blood brain barrier via increased permeability [5,6]. To elucidate the mechanisms of this disruption, the authors note that induction of angiopoietin-like 4 did not impact expression of tight junction proteins but increased expression of MYL5, which in turn, activated the RhoA signaling pathway and displayed a negative role on barrier function regulation.

A second contribution from the Wang and Chen laboratories led by Amjad et al., built upon their expertise in meningitic *E. coli* by investigating the regulation of severe neuroinflammatory damage at the blood brain barrier during infection [7]. Built upon their previous transcriptional profiling of microRNA (miRNA) expression in brain microvascular endothelial cells following bacterial meningitic infection, the authors defined the downregulation of a miRNA, miR-19b-3p using in vitro and in vivo models [5,8]. These studies revealed a role for the miRNA in attenuation of proinflammatory cytokine production and chemokines through increased expression of TNFAIP3, a negative regulator of NF-κB signaling, and exogenous injection of the miRNA aggravated the inflammatory damage. Taken together, this work proposes a host mechanism to quench neuroinflammatory damage associated with bacterial meningitis through regulation of miRNA expression and the signaling targets.

This Special Issue also covers the role of microbial pathogenesis with thought-provoking Reviews spanning diverse species of bacterial and fungal pathogens. For example, the complexity of Lyme disease in consideration of the illness burden, incidence rates, consequences of infection, and optimal case management strategies are explored by Bamm et al. to highlight the intricate relationship between host and pathogen during this disease [9]. This extensive Review discusses the juxtaposition between established and emerging concepts in *Borrelia* (the causative agent of Lyme disease) biology and pathogenesis through coverage of diagnostic challenges and disease classification, diverse components of the host-pathogen interplay that impact disease severity and outcome, as well as the consequences (e.g., resistance) and opportunities (e.g., alternative anti-microbials) with medical intervention. The Review also extends into presentation of high-priority issues raised by patients and clinicians, as well as recommendations for addressing diagnostic uncertainties and intervention protocols for the effective and long-lasting treatment of Lyme disease.

Among bacterial pathogens, the Gram-negative *Pseudomonas aeruginosa* has been extensively studied in the context of biofilm formation and the role of cell wall components contributing to host evasion. For example, Geddes-McAlister et al. discussed the limited efficacy of host immune cells, specifically, neutrophils in clearing bacterial biofilms [10]. Here, the authors present the role of the essential components of *P. aeruginosa* biofilms, including polysaccharides, alginate, and extracellular DNA and their roles in protecting the bacteria from neutrophil attack. The authors also discuss opportunities for enzymatic degradation of bacterial biofilms to promote susceptibility to antimicrobial therapy and enhance immune cell clearance for effective treatment options [11,12,13]. Moreover, Huszczynkski et al. highlight the contradictory roles of *P. aeruginosa* lipopolysaccharide (LPS) in host cell recognition and bacterial survival within the host environment [14]. The authors present the diverse roles and unique properties of LPS in the context of structure and organization, interactions with the host immune system, influence on bacterial physiology, and the ability of antimicrobials to target LPS for effective treatment of bacterial infection. As discussed by the authors, the biosynthesis of LPS represents a promising target for therapeutic development; however, the major hurdle of resistance and the evolution of bacterial pathogens to adapt the structure and composition of drug targets must be considered when exploring new drug options. Furthermore, recent advances towards the production of bacterial vaccines holds promise for a prophylactic measure against the invading pathogens [15].

The intricate relationship between host and pathogen involves many key players with distinct roles for protection, which may be directly or indirectly influenced by the type of pathogen encountered. For example, Kretschmer et al. describe the vital role of chloroplasts in plant immunity through the synthesis and production of secondary metabolites and defense compounds, as well as phytohormones in response to invading pathogens [16]. A plethora of studies have described the role of chloroplasts in protecting plants from viral and bacterial infections; however, only recently, the role of fungal effectors destined for the chloroplast have been reported. Here, the authors extrapolate on well-characterized chloroplast-targeting bacterial effectors as evidence for fungal effectors with similar roles in chloroplast attacks. As intended, the contributions to this Special Issue cover a diverse array of biological systems with broad relevance to health and agricultural settings. The presented works highlight similarities between organisms and provide an opportunity to draw on information gleaned from one organism, species, or kingdom to address questions in a related field to present novel and complementary approaches to surviving and combatting microbial pathogenesis.
